# Anæsthetics

**Published:** 1893-07

**Authors:** 


ARTICLE VII.

### ANÆSTHETICS.

In the early days of my practice I had a novel experience in extracting several teeth. A stalwart farmer (at least in physique) who could tip the scales at fully three hundred, but more timidity could not possibly have been crowded into the same amount of avoirdupois. He had been in my chair before, I *knew* him. With almost herculean effort he could face the music for one tooth, but when it came to eight or ten it was too much for his peculiar make-up. He took the chair with dreadful anticipations, and many exclamations, directions, etc., etc. I advised him to lie back in the chair and keep quiet; then I began to lay out the forceps, at the sight of which he fainted. He soon was as blue as indigo and as limp as a rag. My first thought was for restoratives, but my presence of mind saved me and I resolved to avail myself of an extempore anæsthetic, and out came the teeth quicker than the time taken to write this. Looking at him still in a deadly faint, the blood pouring, I could scarcely realize myself what I had been doing. Then I commenced with my restoratives, dashed cold water in his face and put the bottle of ammonia to his nose. I soon had the satisfaction of seeing his eyes rolling about, but dazed, confused; and not until he saw the blood running over the linen did he take in the situation, and immediately his tongue went on a voyage of discovery; and when he found his teeth all out he was a happy mortal. It is the only instance of the kind in all my long experience of fifty years.

From actual practice of nearly half a century with the various anæsthetics that have been in use, both by local application, inhalation and by hypodermic injection, I propose briefly to give your readers my observation upon the best anæsthetics in use, the manner of administering, and a few hints about inhalers and the management of patients. In 1847 my attention was brought to the use of ether (which antedates chloroform), the inhalation of which would produce complete unconsciousness, and teeth could be extracted without pain. I immediately ordered a bottle of ether and an inhaler from Buffalo, accompanying which came instructions for the administration, which I thoroughly mastered, and then was looking out for a patient, whom I soon found in the person of an itinerant singing master. Being ambitious and anxious to introduce this new method, I convened the notables of the town (Belleville) where I was then in practice—the mayor, the sheriff, barristers, doctors, etc. It being the first attempt, and unacquainted with the effect it might produce upon my patient, I candidly admit it was not without some misgivings, but I nevertheless put on a wise look and appeared self-possessed, which instilled confidence in my auditors. The operation was a perfect success, patient delighted, my auditors satisfied, and I proud of my achievement. An intelligent chemist and druggist who had quite a reputation in extracting teeth, remarked, "He was disgusted with his manner of extracting, and would henceforth give it up," which he did, and I soon had the entire field for fifty miles north, south, east and west. Soon after the advent of ether came in chloroform, that great rival which soon put ether in the shade. Ignorant of its dangerous character, I administered it indiscriminately for many months, indeed, became "the chloroformist"—administered it for physicians in important surgical operations. Like a thunder-clap out of a clear sky came the report that a man had died in a dentist's chair at Toronto from the administration of chloroform. I never after ventured to give it without a physician being present. Here let me say that during the administration of any anæsthetic, absolute silence should be maintained. There should be no talking save the gently repeated request of the administrator to the patient to keep his eyes shut and take long breaths.

Before administering any anæsthetic, educate your patient. While preparing the inhaler, address your patient after this manner: "You have some bad teeth there; they must have given you a good deal of pain." (Next allow your patient to talk.) "You are here to get rid of them, and I am anxious to relieve you, but I cannot do it unless you assist me. You may be somewhat anxious, but it will take away the keenness of the pain, and remember to open your mouth, and I will do my best." Since adopting this method I have been sometimes astonished at the success. In the administering of ether, no inhaler should be used the second time. Extemporize one by forming a cone of paper or a towel, and drop in a *clean* piece of sponge. When a mother or father comes to your surgery with a child, say five years of age, to have some temporary teeth or teeth extracted, and must be quieted with something, direct the parent to take the chair and hold the little patient in his arms. Then talk about the weather, etc., and at the same time pour a little chloroform upon a handkerchief and commence to pass it back and forth. Don't mind if the parent gets a little. Soon the child will be sufficiently under the influence to extract all you desire.

When a patient, say between the age of eight and ten, comes with a six-year molar aching, I do not hesitate to give them chloroform administered from a common tumbler. Drop in a napkin and pour on a *little* chloroform at first, again a little and you will have no difficulty in accomplishing the object. I have no fear in using it for children. Of course I would not give it to a sickly child. My only diagnosis is observation and a few questions put to parents or guardians.

The most eminent surgeons who have lost patients under the influence of chloroform, evidently failed in their diagnosis, and hence it follows that it is never safe, especially in adults, and should never be given without great care. It is not a question, but an established fact, that there is no means of recognizing the idiosyncrasy except by the actual administration of the drug, and that death is the first intimation of its existence, and that no precaution on the part of the administrator will suffice to avert the fatality. Ether is the agent now in general use, and has nearly if not quite supplanted chloroform, though I never have used it in my practice without the assistance of some qualified physician, though many eminent medical men say it never was the *direct* cause of death. Several fatal cases have been reported from the administration of nitrous oxide, but just how reliable is a question. Chloroform, ether, and nitrous oxide are the only standard agents now in use by the dental profession when inhalation is advised. I copy the following from the *Cosmos* of March, 1876:

"Surgical Anæsthesia in Children. This is the method of procedure at the Hopital des Enfans Malades: At 8 o'clock the Sister administers from three to four grammes (according to the age of the child) of chloral, and the child goes to sleep in twenty minutes. At nine o'clock the dentist passes through the ward and pulls the tooth, at times two teeth, and when the child wakes up, after three or four hours, it is minus its tooth, without suffering, or having seen the dentist."

Those who know the pain of having a tooth extracted, and the difficulty of doing this with children, will appreciate in chloral a precious agent.

Local anæsthesia is now engaging the attention of the profession, which I may consider in my next.—*Dominion Dental Journal.*

